# Some Good Things That Are to Appear in the Red Back

**Published:** 1914-12

**Authors:** 


### Some Good Things That are to Appear in the Red Back.

Editorial, Antisepsis and Asepsis, Dr. George H. Lee, Galveston, Texas.

A paper on Pellagra, by Dr. Wilmer Allison, of Fort Worth.

Two articles on Artificial Pneumothorax, by Dr. A. G. Shortle, of Albuquerque, N. M. So far the doctor has treated 82 cases. In the first article he will go into the history of the case, describe the operation, etc. In the second article he will go into details as to results, complications, etc.

A thrilling account of the difficulties encountered by Andreas Vesolius in his effort to study the anatomy of the human body. Introduction by Dr. A. W. Fly, of Galveston, Texas.

Alcohol and the Public Health, by Dr. J. N. Hurty, State Health Commissioner of Indiana.

The Use of the Obstetrical Forceps, by T. J. Turpin, of Corpus Christi, Texas.

An article by Dr. L. P. Tenney, of Troup, Texas.

A paper on the work being done by the Malthusian League, by Charles V. Drysdale, Hon. Secretary, Westminster, London, England.

A paper by Dr. L. W. Crosthwaite, of Waco, Texas.

